# Book Reviews

**Published:** 1873-02

**Authors:** 


### BIBLIOGRAPHICAL.

---

*A System of Oral Surgery:*—Being a consideration of the diseases and surgery of the mouth, jaws, and associate parts. By James E. Garretson, M. D., D. D. S. Illustrated with numerous steel plates and wood cuts. Published by J. B. Lippincott & Co., Philadelphia, 1873.

This valuable work is a second edition of that published in 1869, under the title of "Diseases and Surgery of the Mouth, Jaws, and Associate Parts," which contained forty-two chapters, while this second edition contains fifty-one; commencing with a student's preface defining disease, which, the author remarks, in the greatest breadth, as in the narrowest of its signification, has its meaning in a single word,—"irritation." This second edition has been greatly modified and enlarged, and the treatment of dental caries brought up to its present status. We considered the first edition a valuable contribution to dental literature, and of great service to the student and dental practitioner, and therefore receive with considerable satisfaction, this modified and enlarged one. The object of the author is to so instruct the dental student, that he will be qualified to undertake and successfully treat all diseases connected with the mouth, jaws, and associate parts, and he has succeeded in presenting a work which will prove of incalculable value to all engaged in the practice of dentistry.

---

*Herald of Health.*—The February number contains a most able and instructive paper, translated from the French, on the Hygiene and Physiology of Marriage, which every person, old or young, should read. It is high toned, and will do great good. Also Tobacco—Its effects on the Human Constitution, Physical, Intellectual and Moral," by J. C. Layard, M. D.; "California as a Resort for Invalids;" "Care of Fretful Babies;" the story of "Franklin's Whistle," as told by himself, and some forty or fifty other articles.

This Journal contains so many topics of daily interest, as to render it an instructive and useful publication to all classes in life. Wood & Holbrook, Publishers, 15 Laight Street, N. Y. Subscription, $1.50 per year.

*Vick's Illustrated Floral Guide for* 1873 has been received, surpassing, if possible, the former editions in beauty and profuseness of illustrations.

So enterprising and energetic is Mr. Vick, that his name has become famous abroad, as well as at home, as one of the first florists of the age. His beautiful "Floral Guide" is now published quarterly, and will contain valuable information for the cultivator of both flowers and vegetables, each of the four numbers being adapted to the season in which it appears. It is published in German as well as in English. The small sum of twenty-five cents pays for the year, which is not one-third of the cost. Jas. Vick, Publisher, Rochester, N. Y.

